# Action Mechanism of *Ginkgo biloba* Leaf Extract Intervened by Exercise Therapy in Treatment of Benign Prostate Hyperplasia

**DOI:** 10.1155/2013/408734

**Published:** 2013-04-16

**Authors:** Chiung-Chi Peng, Jia-Hong Liu, Chi-Huang Chang, Jin-Yuan Chung, Kuan-Chou Chen, Kuang-Yu Chou, Robert Y. Peng

**Affiliations:** ^1^Graduate Institute of Clinical Medicine, College of Medicine, Taipei Medical University, 250 Wu-Hsing Street, Taipei 11031, Taiwan; ^2^Research Institute of Biotechnology, Hungkuang University, 34 Chung-Chie Road, Shalu District, Taichung City 43302, Taiwan; ^3^Department of Urology, Shuang Ho Hospital, Taipei Medical University, 291 Zhongzheng Road, Zhonghe, Taipei 23561, Taiwan; ^4^Department of Urology, School of Medicine, College of Medicine, Taipei Medical University, 250 Wu-Hsing Street, Taipei 11031, Taiwan; ^5^Division of Urology, Department of Surgery, Shin Kong Wu Ho-Su Memorial Hospital, 95 Wen Chang Road, Taipei 111, Taiwan

## Abstract

Benign prostatic hyperplasia (BPH), an imbalance between androgen/estrogen, overexpression of stromal, and epithelial growth factors associated with chronic inflammation, has become an atypical direct cause of mortality of aged male diseases. *Ginkgo* possesses anti-inflammatory, blood flow-enhancing, and free radical scavenging effects. Considering strenuous exercise can reduce BPH risks, we hypothesize *Ginkgo* + exercise (*Ginkgo* + Ex) could be beneficial to BPH. To verify this, rat BPH model was induced by s.c. 3.5 mg testosterone (T) and 0.1 mg estradiol (E2) per head per day successively for 8 weeks, using mineral oil as placebo. Cerenin^®^ 8.33 **μ**L/100 g was applied s.c. from the 10th to the 13th week, and simultaneously, Ex was applied (30 m/min, 3 times/week). In BPH, *Ginkgo* alone had no effect on T, 5**α**-reductase, and dihydrotestosterone (DHT), but suppressed androgen receptor (AR), aromatase, E2 and estrogen receptor (ER), and the proliferating cell nuclear antigen (PCNA); Ex alone significantly reduced T, aromatase, E2, ER, AR, and PCNA, but highly raised DHT. While *Ginkgo* + Ex androgenically downregulated T, aromatase, E2, and ER, but upregulated DHT, AR, and PCNA, implying *Ginkgo* + Ex tended to worsen BPH. Conclusively, *Ginkgo* or Ex alone may be more beneficial than *Ginkgo* + Ex for treatment of BPH.

## 1. Introduction

Benign prostatic hyperplasia (BPH) and low urinary tract symptoms (LUTS) are quite common male diseases. The prevalence of BPH increases with aging [1] and has now become an atypical direct cause of mortality [2]. Biochemically, BPH is considered to be an imbalance between androgen/estrogen [3, 4], overexpression of stromal and epithelial growth factors, cytokines, and steroid hormones [5, 6]. Pathologically, BPH is characterized by hyperplastic epithelial and stromal growth that emerge into numerous microscopic and macroscopic nodules in the prostate gland [7]. Tissue remodeling in the aging prostate [8, 9], stem cell defects [10], hypoxia [11], and chronic inflammation [12–16] or by many other factors is still obscure.

The clinical care for BPH usually involves *α*-blockers, 5*α*-reductase inhibitors (e.g., finasteride), and surgery therapy, or the combined treatment [17]. Currently, phytotherapeutic agents are emerging and frequently used as the complementary alternative treatment of BPH [18]. Other nonmedication agents include zinc, soy/tofu, selenium, vitamin E, and amino acids [19]. The main constituents of *Ginkgo biloba* leaf extract (for simplicity named “*Ginkgo*” herein) comprise *Ginkgo *flavone glycosides (biflavonols, quercetin; biflavones, sciadopitysin; and proanthocyanidins, procyanidin) and terpene lactone ginkgolides (ginkgolide A, B, C, and bilobalide) [20]. *Ginkgo *possesses antioxidant and anti-inflammatory activity [21–24]. *Ginkgo biloba* leaf extract (*Ginkgo*) has been used for centuries in China for treating asthma and bronchitis. Currently, *Ginkgo* has been widely used to treat the cerebrovascular and the peripheral vascular insufficiency, neurosensory problems, and disturbances in vigilance, short-term memory, and other cognitive functions that are associated with dementias, ageing, and senility [25]. Many pharmacological and clinical studies have shown that the extract of *Gingko *causes two main actions: increase of blood flow in central and peripheral vessels and inhibition of platelet aggregation and free radical scavenging. Thus *Gingko *may be effective in cases of erectile dysfunction due to a decreased blood flow [26].

A review of Sea et al. (2009) highly supports a clinically significant, independent, and strong inverse relationship between exercise (Ex) and the development of BPH /LUTS [27]. Greater distances run per week may reduce BPH risk independent of BMI, 10 km performance, and diet [28].

The application of *Ginkgo *in treating BPH is still lacking. In view of the above mentioned beneficial biological activities of *Gingko *and Ex, we hypothesize that *Ginkgo* + Ex therapy as well could be beneficial to BPH. To verify this, we conducted this experiment and the relevant biochemical, immunological, and pathological parameters were examined and compared.

## 2. Materials and Methods

### 2.1. Chemicals

Testosterone, dihydrotestosterone (DHT), estradiol ELISA kits were provided by Cayman Chemical Co. (Michigan, USA). Free PSA and Total PSA assay kits were provided by Cusabio Biotech (Wuhan, China). Rat IL-1 ELISA development kit is product of PeproTech Co. (Rocky Hill, NJ, USA). Enhanced Chemiluminescence (ECL) system was provided by Merk Millipore Co. (Billerica, MA, USA). TEMED is a product of Bio-Rad Co. (Hercules, CA, USA). Protein Extraction Solution was provided by iNtRON Biotech. Co. (Kyungki-Do, Korea). The pharmaceutical preparation of androgen, Sustanon^®^, is an inject testosterone medication provided by Schering-Plough Company (Kenilworth, NJ, USA), which in reality contains four testosterone esters. Each ampoule (1 mL) contains testosterone propionate 30 mg, testosterone phenylpropionate 60 mg, testosterone isocaproate 60 mg, and testosterone decanoate 100 mg. The overall androgenic potency in per mL of Sustanon^®^ is equivalent to 176 mg testosterone. The *Ginkgo biloba *leaf extract, Cerenin^®^, was purchased from Dr. Willmar Schwabe Arzneimittel GmbH & Co. (Karlsruhe, Germany). *Ginkgo *consisted of 24% of *Ginkgo *flavone glycosides, mainly kaempferol and quercetin glucorhamnoside esters, and 6% of the characteristic terpene lactones, the bilobalide, and ginkgolides, namely, A, B, C, and very small quantities of ginkgolide. The inject solution contained in per mL 3.5 mg *Ginkgo *(240 mg/g flavonoids and 60 mg/g terpenoids), 30 mg of 960 mL/L ethanol, 40 mg sorbitol, and 0.1 mol/L NaOH. All other chemicals, not cited but used in this experiment, were of reagent grade provided by Wako Pure Chemical Co. (Osaka, Japan). The sources of the antibodies used in this experiment were 5*α*-reductase and androgen receptor (Santa Cruz Biotech Inc., Santa Cruz, CA, USA), estrogen receptor *α* (ER*α*) (Merk Millipore, Billerica, MA, USA), aromatase and proliferating cell nuclear antigen (PCNA) (Epitomics Inc., Burlingame, CA, USA), and *β*-actin from Novus Biologicals (Littleton, CO, USA).

### 2.2. Animal Grouping

This experiment was proved by the Institutional Animal Care and Ethic Committee of China Medical University (Taichung, Taiwan). Ninety-six Sprague-Dawley (SD) rats weighing approximately 280 g were purchased from BioLasco Animal Centre, Taiwan. The rats were housed in a controlled environment, 3 in each cage, with 12 h/12 h light/dark cycle, at 28 ± 1°C and under relative humidity 65–75%. These rats were acclimated in such an environment for the first week. Then rats were divided into eight groups. The five controls involved Group 1, the normal control; Group 2, the BPH control; Group 3, the *Ginkgo*-only treated control; Group 5, the Ex-only control; and Group 7, the *Ginkgo* + Ex. The medication treated groups were Group 4, BPH treated with *Ginkgo*; Group 6, BPH treated with Ex; and Group 8, BPH treated with *Ginkgo* + Ex, each having 12 rats. The exercise groups were subjected to treadmill exercise protocol. The treatment started from week 10 to week 13.

### 2.3. BPH Induction: The Hormone-Induced Rat BPH Model

The protocol to induce BPH was conducted according to Suzuki et al. (1994) with slight modification [29]. Briefly, in the beginning of week 2, all healthy controls (groups 1, 3, 5, and 7) were s.c. administered 20 *μ*L mineral oil/head/day as placebo. The BPH groups (groups 2, 4, 6, and 8) were s.c. administered a combined testosterone (Sustanon^®^) 3.5 mg with estradiol 0.1 mg per head per day successively from week 2 to week 9, that is, a total induction period of 8 weeks.

### 2.4. *Ginkgo biloba* Extract Administration Protocol

The dose of Cerenin^®^ was calculated from the recommended dose for clinical human use, that is, 35 mg (5 mL injection solution) i.v. for 60 kg adults. Thus in the beginning of week 10 immediately prior to the treatment experiment, a dose of Cerenin^®^ 8.33 *μ*L/100 g was recommended daily for each *Ginkgo *medicated group. The whole treatment course sustained for 4 weeks (i.e., from week 10 to week 13).

### 2.5. Treadmill Exercise Training Protocol

For treadmill exercise training, the groups 5, 6, 7, and 8 were first acclimated from week 8 to 9, starting with 5 min, 10 min, 15 min, and then 20 min per time per day. The formal exercise therapy was then started from week 10 until week 13, three times per week, 30 min per time at a speed of 30 m/min on a motorized rodent treadmill (Fortelice International Co., Ltd., Taipei, Taiwan).

### 2.6. Blood and Tissue Collection

At the end of week 13, blood samples were withdrawn from the abdominal aorta under ether anesthesia. The blood was collected and centrifuged at 3000 ×g for 15 minutes to separate the serum. The prostates were excised, immediately frozen with liquid nitrogen and stored in −80°C or fixed by immersion with 10% formalin in PBS (pH 7.4).

### 2.7. Hematoxylin-Eosin (HE) and Sirius Red Staining

The prostates were fixed by immersion in 10% formalin-PBS (pH 7.4) at 4°C for 24 h and processed for paraffin embedding. Paraffin sections were dewaxed in xylene and rehydrated in a series of ethanol washes. The nuclei of these specimens were subjected to hematoxylin-eosin stain. Otherwise, the collagen content was stained with Sirius Red (Sigma-Aldrich Co., MO, USA) [30].

### 2.8. Enzyme-Linked Immunosorbent Assay (ELISA)

Serum levels of testosterone, estradiol, dihydrotestosterone (DHT), prostate-specific antigen (PSA), and IL-1 were measured by the ELISA kits. All protocols were performed by following the manufacturer's instruction. The EZ Read 400 Microplate Reader used was a product of Biochrom Co. (Cambridge, UK) [30].

### 2.9. Immunohistochemical (IHC) Staining

The protein expression of androgen receptor and proliferating cell nuclear antigen were analyzed by IHC according to previous protocol cited [30]. Quantitative analysis was performed using an Image-Pro Plus (Meyer Instruments, USA) analysis system. The integrated optical density (IOD) was measured. The sum of the IOD was obtained with the mean value calculated.

### 2.10. Western Blotting

Levels of 5*α*-reductase, estrogen receptor *α*, and aromatase were analyzed. Briefly, frozen prostate tissue samples (approximately 100 mg) were homogenized with the homogenizer (T10 basic, The IKA Company, Germany) in 1 mL of Pro-PREP lysis buffer (pH 7.2). The homogenate was centrifuged at 12000 ×g for 20 min at 4°C, and the supernatant was collected as tissue sample lysate. The sample lysates were heated at 100°C for 10 min before loading and separated on precasted 7.5% SDS-PAGE. The proteins were electrotransferred onto the PVDF membrane in transfer buffer for 1 h.

The nonspecific binding to the membrane was blocked for 1 h at room temperature with 5% nonfat milk in TBS buffer. The membranes were then incubated for 16 h at 4°C with various primary antibodies. After extensive washing in TBS buffer, the membranes were then incubated with secondary antibody in blocking buffer containing 5% nonfat milk for 1 h at room temperature. Membranes were then washed with TBS buffer and the signals were quantified using the Luminescent Image Analyzer LAS-4000 (Fujifilm, Tokyo, Japan). *β*-actin was used as the reference protein [30].

### 2.11. Statistical Analysis

Data obtained in the same group were treated with ANOVA and Duncan's multiple range tests with computer statistical software SAS 9.0 (SAS Institute Inc., Cary, NC, USA). Different letters indicate significant differences at *P* < 0.05.

## 3. Results

### 3.1. BPH Reduced the Body Weight but Raised the Ratio of Prostate Weight to Body Weight

**Figure 1:**
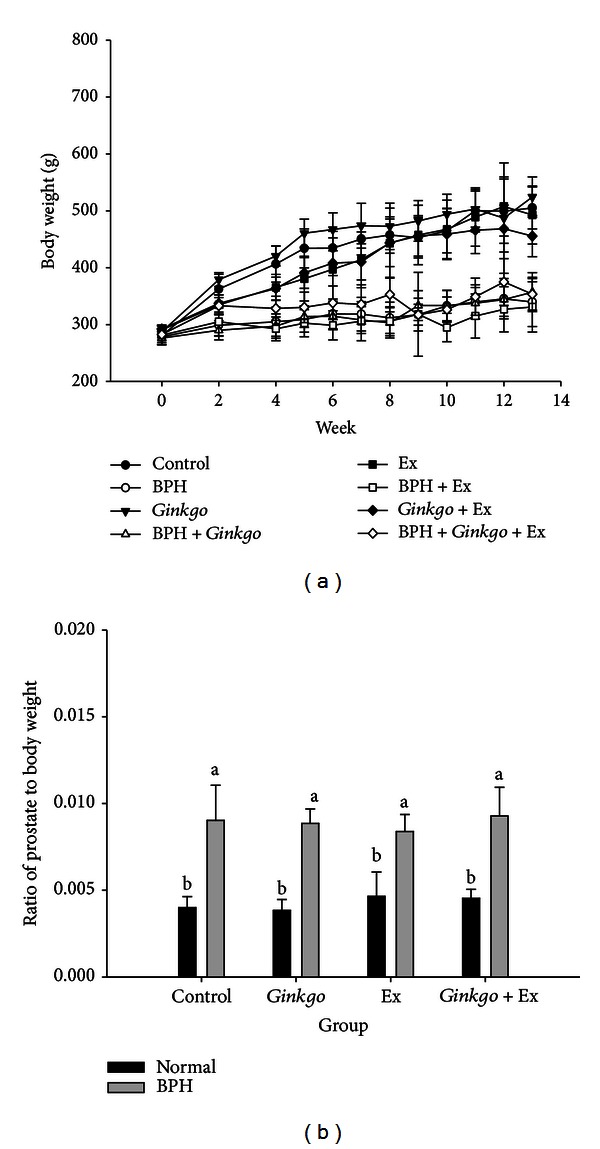
Duration-dependent variation of body weight (a) and the ratio prostate to body weight among all groups (b). Week 0-week 1: acclimation. Week 2–week 8: BPH induction period. Week 9: exercise pretraining. Week 10–week 13: receiving treatment. The grouping of rats was described in the text. (12 rats in each group, *P* < 0.05) BPH: benign prostate hyperplasia. Ex: exercise.

Starting from the original body weight (275 to 300 g), the body weight of all groups was seen increasing steadily from week 0 to week 2 (week 1 for acclimation) (Figure 1(a)). When induced with BPH at week 2 and with induction maintained daily until week 9, all control groups showed substantive increase of body weight to range within 455.5 ± 36.2 ~ 524.4 ± 35.2 g at week 13. As contrast, in all BPH groups no apparent body weight increase was found; all remained in range 331.0 ± 44.2 ~ 356.9 ± 34.2 g/rat until week 13 (Figure 1(a)). The ratio prostate to body weight remained within 0.004 ~ 0.005 in all normal groups but were raised to 0.008 ~ 0.009 in all BPH rats (Figure 1(b)).

### 3.2. Microscopic Pathological Examination

**Figure 2:**
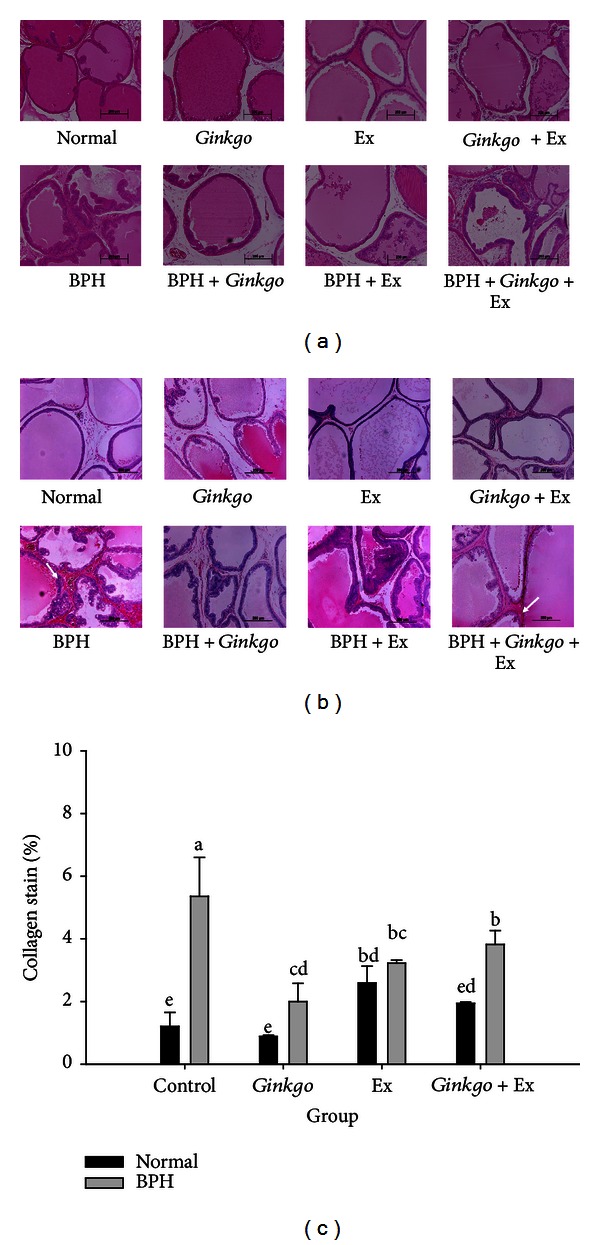
Hematoxylin-eosin stain (a) and Sirius Red stain of collagen deposition (b) of prostatic tissues of the normal controls (upper panel), and the BPH groups (lower panel) (×200); and the quantification of collagen deposition (c). (Upper panels: normal, *Gingko* control, Ex control, and *Gingko* + Ex control; lower panels: BPH control, BPH + *Gingko*, BPH + Ex, and BPH + *Gingko* + Ex.) Data were collected and statistically treated with ANOVA and Duncan's multiple range tests (*n* = 3). Different letters indicate significant difference between groups (*P* < 0.05). BPH: benign prostate hyperplasia. *Gingko*: the *Gingko biloba* leaf extract. Ex: exercise.

Microscopically, in the normal prostates the acini were lined by columnar epithelial cells and the lumens were filled with eosinophilic secretion. Regular acini and alignment were apparently perceivable (Figure 2(a), upper panel). In the BPH prostates, mild epithelial hyperplasia and irregular acinar shape with villous projections were clearly perceived. The epithelial hyperplasia budding out with intraepithelial vacuoles was very apparent, and in this region some epithelial cells indicated loss of polarity (Figure 2(a) lower panel). Statistically, the pathological incidence rate (rat number per group) of prostate hyperplasia was 0.00, 0.73, 0.00, and 0.18 for the four groups without exercise, that is, the normal control, BPH control, *Ginkgo *control, and BPH + *Ginkgo*, respectively. When treated with exercise, the incidence rates were apparently shifted to 0.00, 0.00, 0.00, and 0.18, respectively, for Ex control, BPH + Ex, *Ginkgo* + Ex, and BPH + *Ginkgo* + Ex. Usually BPH is accompanied with inflammation. The incidence rate of inflammation was 0.00, 0.64, 0.08, and 0.18, for the normal control, BPH control, *Ginkgo *control, and BPH + *Ginkgo*, respectively. After exercise training, the incidence rate of inflammation was improved to give 0.00, 0.46, 0.25, and 0.55, respectively, for Ex control, BPH + Ex, *Ginkgo* + Ex, and BPH + *Ginkgo* + Ex (Drs. Chen T.-Y. and Lee K.-H., Diagnostic Laboratory of Laboratory Rodents, National Laboratory Animal Center (NLAC), Taipei, Taiwan), implying the fact that *Ginkgo *was beneficial to curing BPH, and also effective to suppress the inflammation status. The Sirius Red stain revealed huge amount of collagen deposition occurring in BPH group (Figures 2(b) and 2(c)), apparently localized in the interstitial tissues and intracellular cytoplasm. *Ginkgo *alone seemed to have alleviated the majority of these pathologically adverse effects. Exercise alone improved it to some extent only comparable to the effect with *Ginkgo* + Ex (Figures 2(b) and 2(c)). Amazingly, the normal Ex groups also revealed some collagen deposition (Figures 2(b) and 2(c)).

### 3.3. Serum Total Prostate-Specific Antigen (*t*-PSA) in BPH Was Upregulated by *Ginkgo*, but Suppressed by Exercise Training

**Figure 3:**
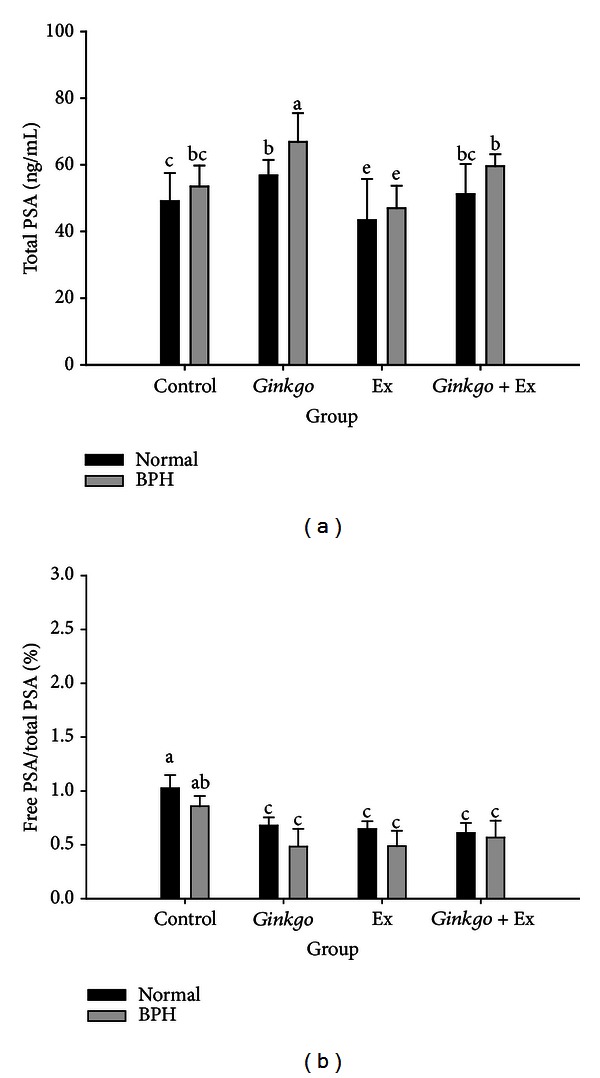
Serum level of total-PSA (a) and the percent ratio free-to-total PSA (b), of the normal controls and the BPH groups when treated with *Gingko*, Ex, and *Ginkgo* + Ex. Data were collected and statistically treated with ANOVA and Duncan's multiple range tests (*n* = 3). Different letters indicate significant difference between groups (*P* < 0.05). PSA: prostate-specific antigen. BPH: benign prostate hyperplasia. Ex: exercise.

The normal total PSA level of the control and the BPH groups were ranging within 44 ± 8 ng/mL and 55 ± 5 pg/mL (Figure 3(a)). *Ginkgo *elevated the levels in both the *Ginkgo* and the BPH + *Ginkgo *groups significantly to 57 ± 6 pg/mL and 68 ± 9 pg/mL. As contrast, exercise significantly reduced the total PSA levels in both the Ex and BPH + Ex groups. Conversely, in the *Ginkgo* + Ex group, both levels remained unchanged when compared with the normal and BPH control groups (Figure 3(a)).

### 3.4. Free PSA/Total PSA Ratio Was Suppressed by *Ginkgo*, Exercise, and the Combined Therapy

The free PSA to total PSA ratio (*f*-PSA/*t*-PSA) in normal controls ranged within 1.03 ± 0.12%, and that of BPH within 0.86 ± 0.10%. *Ginkgo*, exercise, and *Ginkgo* + Ex all significantly reduced the levels to ranges within 0.48 ~ 0.68 pg/mL (Figure 3(b)). No apparent difference was found for any treated group, indicating the effect of *Ginkgo *to be comparable to exercise alone. However in the combined therapy of *Ginkgo* + Ex, no better effect was further found (Figure 3(b)); suggestively, the action mechanism of *Ginkgo *and exercise probably via the same pathway.

### 3.5. The Serum Level of Testosterone Was Not Affected by *Ginkgo* Alone, but Improved Slightly by Exercise and the Combined Therapy

**Figure 4:**
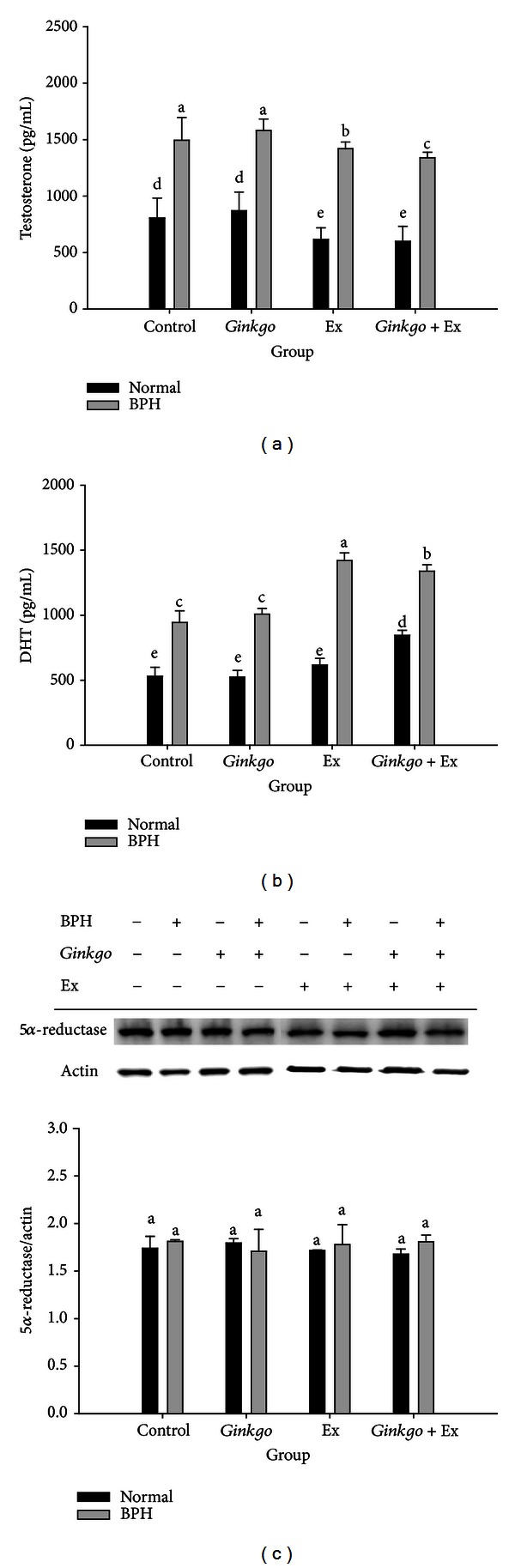
Serum levels of Testosterone (a), DHT (b), and the Western blotting of 5*α*-reductase (c) in prostates of the normal controls and the BPH groups. *β*-actin was used as the reference constitutive protein. Data were collected and statistically treated with ANOVA and Duncan's multiple range tests (*n* = 3). Different letters indicate significant difference between groups (*P* < 0.05). DHT: dihydrotestosterone. BPH: benign prostate hyperplasia. Ex: exercise.

The serum level of testosterone in the normal control maintained at a level of 806.8 ± 175.6 pg/mL. While in the BPH control, it was apparently raised to 1494.1 ± 202.1 pg/mL. Exercise alone and *Ginkgo* + Ex seemed to have only slightly yet significantly suppressed these testosterone levels (Figure 4(a)).

### 3.6. *Ginkgo* Alone Did Not Affected DHT, However Exercise and the Combined Therapy Significantly Raised DHT Level in BPH Groups

*Ginkgo* alone did not affect the prostatic DHT levels in both the normal and BPH groups. Alternatively, exercise and the combined therapy significantly elevated the prostatic DHT levels to reach 1420.4 ± 58.8 and 1338.7 ± 49.8 pg/mL in BPH groups, respectively (Figure 4(b)). The DHT levels in the normal *Ginkgo* + Ex group also increased (844.64 ± 39.1 pg/mL) compared with normal control (530.8 ±68.3 pg/mL) (Figure 4(b)).

As contrast, Western blotting revealed 5*α*-reductase to be totally unaffected by any of these specific therapies (Figure 4(c)).

### 3.7. AR Highly Upregulated by BPH Was Separately Downregulated by *Ginkgo* and Ex Alone, yet Further Upregulated by *Ginkgo* + Ex Therapy

**Figure 5:**
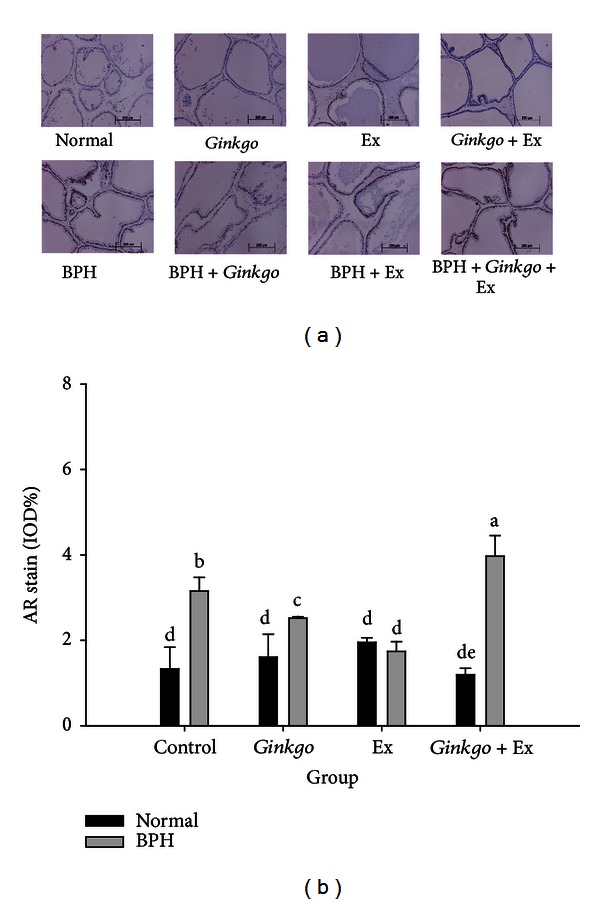
Immunohistochemical stain of AR (a), and its quantification (b) in the prostate tissues of the normal controls and the BPH groups. (Upper panels: normal, *Gingko* control, Ex control, and *Gingko* + Ex control; lower panels: BPH control, BPH + *Gingko*, BPH + Ex, and BPH + *Gingko* + Ex.) Data were collected and statistically treated with ANOVA and Duncan's multiple range tests (*n* = 3). Different letters indicate significant difference between groups (*P* < 0.05). AR: androgen receptor. BPH: benign prostate hyperplasia. Ex: exercise.

Immunohistochemical stain showed prostatic androgen receptors were highly upregulated in BPH group (IOD 3.15 ± 0.32%) (Figures 5(a) and 5(b)). *Ginkgo *or exercise was shown able to suppress most parts of the upregulated AR (IOD 2.52 ± 0.04%, 1.74 ± 0.23%). However strangely, *Ginkgo* + Ex further upregulated the level of AR in BPH group (IOD 3.97 ± 0.48%) (Figures 5(a) and 5(b)).

### 3.8. BPH Highly Upregulated Serum Estradiol Which Was Suppressed by *Ginkgo*, Ex, and *Ginkgo* + Ex

**Figure 6:**
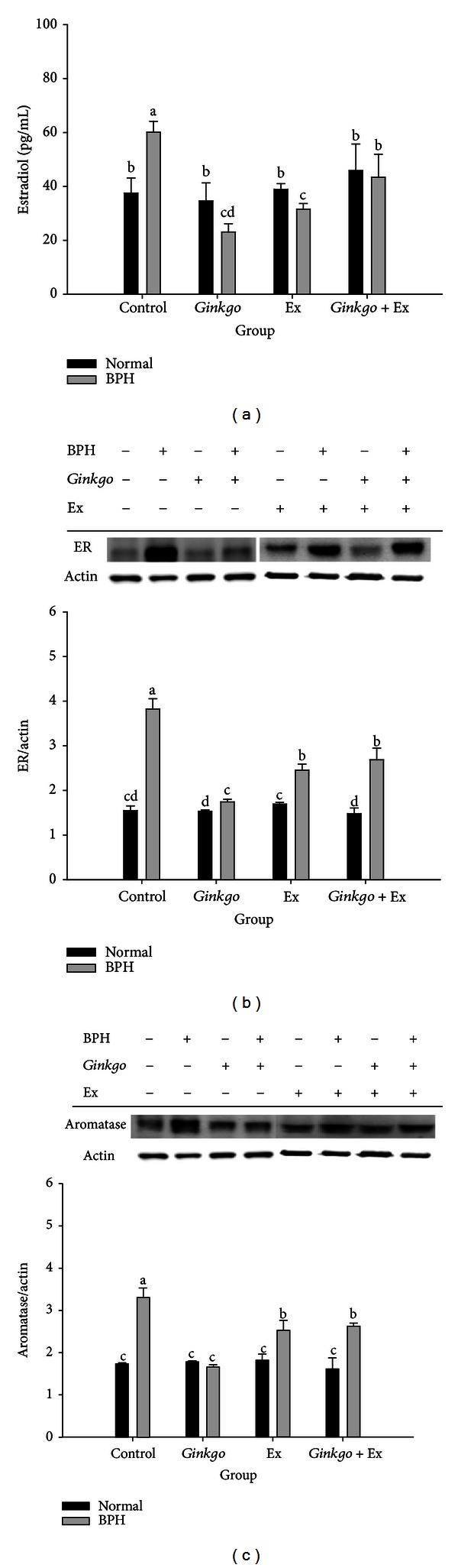
Serum levels of Estradiol (a), the Western blotting of ER ((b) upper) and its quantification ((b) lower), and the Western blotting of aromatase ((c) upper) and its quantification ((c) lower) in the prostates of different groups. *β*-actin was used as the reference constitutive protein. Data were collected and statistically treated with ANOVA and Duncan's multiple range tests (*n* = 3). Different letters indicate significant difference between groups (*P* < 0.05) (*n* = 3). ER: estrogen receptor. Ex: exercise.

Alternatively, the estradiol level of the normal control ranged within 37.5 ± 5.6 pg/mL, and that of BPH control within 60.2 ± 4.0 pg/mL. All three treatments were shown to improve to 23.1 ± 3.1, 31.6 ± 2.0, and 43.4 ± 8.6 pg/mL, respectively, by *Ginkgo*, Ex, and *Ginkgo* + Ex (Figure 6(a)).

### 3.9. The Expression of Estrogen Receptor Was Upregulated by BPH Control, Completely Ameliorated by *Ginkgo*, and Partially Alleviated by Ex Alone and *Ginkgo* + Ex

The levels of estrogen receptor in all normal groups were not affected by any of the three treatments. Conversely, the ER highly upregulated in the BPH group was significantly downregulated by all the three therapies. *Ginkgo *in this case completely alleviated the upregulated level of ER (Figure 6(b)). Alternatively, exercise and the combined therapy seemed to be less effective than *Ginkgo *alone (Figure 6(b)).

### 3.10. *Ginkgo* Suppressed the Aromatase Level of BPH to Better Than Either Ex or the Combined Therapy

Similarly, BPH upregulated prostatic aromatase, *Ginkgo *completely alleviated, and exercise alone and *Ginkgo* + Ex were shown able to partially ameliorate the upregulation of prostatic aromatase. Interestingly, the levels of aromatase in all normal groups were unchanged (Figure 6(c)).

### 3.11. Prostate IL-1, Highly Upregulated in BPH, Was Ameliorated by Any of the Therapies

**Figure 7:**
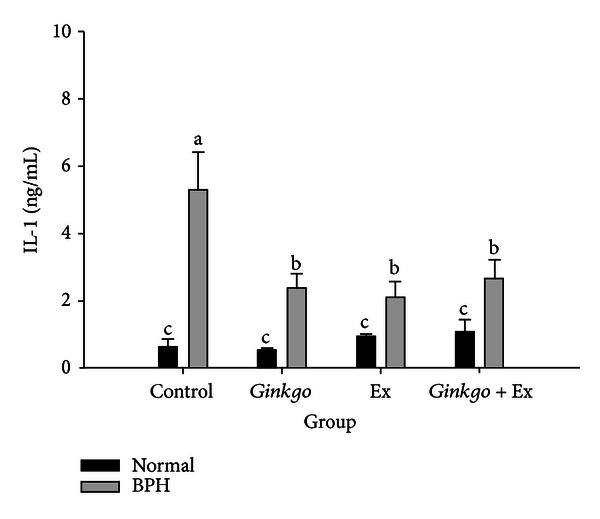
Levels of prostatic tissue interleukin-1 (IL-1) affected by different therapies. Data were collected and statistically treated with ANOVA and Duncan's multiple range tests (*n* = 3). Different letters indicate significant difference between groups (*P* < 0.05).

In prostate of BPH, the level of IL-1 was significantly raised to 5.3 ± 1.2 ng/mL, which was completely ameliorated by all therapies (Figure 7), implying *Ginkgo*, exercise, and the combined therapy to act as effective anti-inflammatory agents.

### 3.12. PCNA Raised in BPH Was Ameliorated by *Ginkgo*, Exercise, and the Combined Treatments

**Figure 8:**
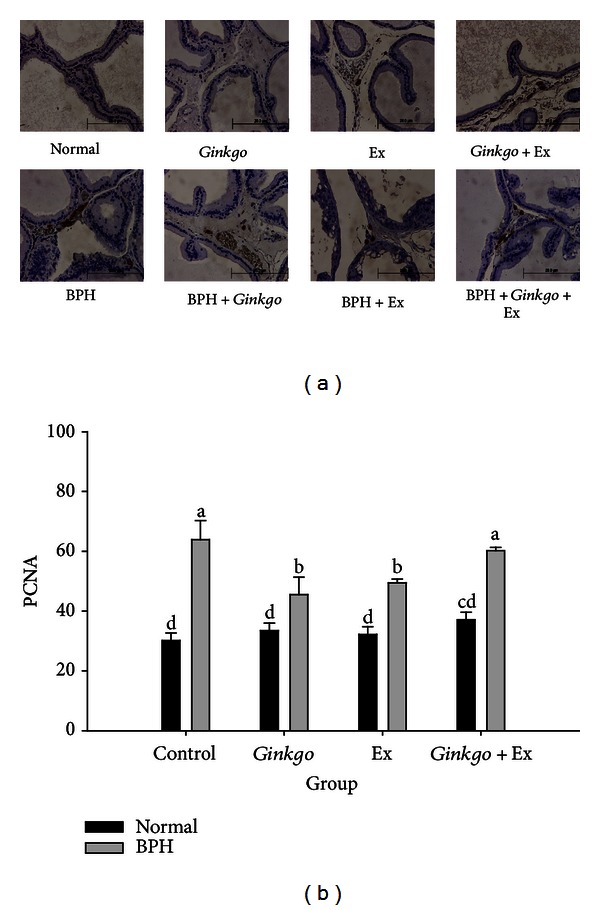
Immunohistochemical stain of the PCNA (a) (×400) and its quantification (b). Data were collected and statistically treated with ANOVA and Duncan's multiple range tests (*n* = 3). Different letters indicate significant difference between groups (*P* < 0.05). PCNA: mouse proliferating cell nuclear antigen.

Either *Ginkgo *or exercise was found to have effectively suppressed the upregulated PCNA in BPH group (Figure 8), apparently showing their promising antiproliferative effect. Astonishingly, the PCNA was insuppressible by the combined therapy (Figure 8). Data were slightly inconsistent with the pathological findings (NLAC).

## 4. Discussions

The normal and all controls showed normal growth increasing rate, conversely the growth rates of all BPH victims were severely retarded (Figure 1(a)). In reality, the body weight and prostate weight can be greatly affected by hormonal status [29]. The castrated male Wistar rats, the castrated + T, and the castrated + T + E2 showed body weight at age 11 weeks 419.6 ± 26.2, 438.0 ± 27.3, and 379.6 ± 26.4 g, respectively, comparing to the normal control body weight 478.6 ± 59.9 g [29]. The body weight retardation in the BPH groups was apparently perceivable. All experimental BPH SD rats retained their body weight within 331.0 ± 44.2 ~ 356.9 ± 34.2 g at week 13 (Figure 1(b)), evidencing the main antigrowth effect exerted by the combined treatment of T + E2.

The effect of *Ginkgo* alone or any of the combined therapy was more or less effective, but limited to some aspects regarding the amelioration of pathological damages in prostates, like the lining up acini by columnar epithelial cells, eosinophilic secretion, epithelial hyperplasia, and deformed acinar shape (Figure 2(a), lower panel). Much of the literatures have indicated many benefits of *Ginkgo*; however most of which were limited only to its antioxidant and anti-inflammatory effects [21–24, 31]. To our belief, we are the first who report *Ginkgo* to be beneficial to BPH. *Ginkgo* was shown to be the most effective with respect to suppressing the intracellular cytoplasmic collagen deposition in the interstitial tissues (Figures 2(b) and 2(c)). Tissue from men with lower urinary tract symptoms was significantly stiffer (*P* = 0.00160) with significantly higher collagen content (*P* = 0.0038) and lower granularity than that from men without lower urinary tract symptoms (American Urological Association symptom index 8 or greater versus 7 or less) [32]. Thus, fibrosis can be a factor contributing to lower urinary tract symptom etiology.

Diagnostically, prostate-specific antigen (PSA) and the ratio of free-to-total PSA are widely used as the tumor markers, but the effect of exercise on these parameters is unclear. Ratio of free-to-total PSA was equally improved by *Ginkgo*, exercise, and *Ginkgo* + Ex (Figure 3(b)). Literature indicated that the free-to-total PSA ratio was significantly lower statistically in master athletes compared with recreational athletes, but is not clinically significant [33]. Nonetheless, the free-to-total PSA ratio can be affected by long-term athletic training, which could be rather important when evaluating athletes with prostate-related disorders [33].

The testosterone levels in groups BPH + Ex and BPH + *Ginkgo* + Ex were seen significantly lower than those in the BPH control. Speculatively, the highly upregulated DHT in these two groups implied BPH possibly incurable by these treatments. AR was seen more highly expressed in BPH + *Ginkgo* + Ex group (Figure 5), while the 5*α*-reductase in all groups remaining at comparable levels, a fact evidently suggesting that exercise and *Ginkgo* + Ex probably were only capable of enhancing the activity but incapable of affecting the quantity of 5*α*-reductase (Figure 4(c)). Suzuki et al. demonstrated that the values of both the *V*_max⁡_ and the *K*_*m*_ for nuclear 5*α*-reductase in the rat dorsal lateral prostate were enhanced by treatment with T + E2 [29]. Thus our findings were rather consistent with Suzuki et al. (1994) [29].

Testosterone is biotransformed into estradiol-17*β* by action of aromatase. The prostate is an estrogen target tissue and estrogens directly and indirectly affect growth and differentiation of prostate [2]. BPH highly elevated the levels of aromatase, estradiol, and ER (Figure 6). Estrogens and selective estrogen receptor modulators have been shown to promote or inhibit prostate proliferation, signifying potential role of BPH and LUTS [2]. Aromatase, estradiol, and ER upregulated in BPH were suppressed by all the three treatments, while *Ginkgo* showed the most promising effect (Figure 6).

*Ginkgo* has been shown to exhibit estrogenic and antiestrogenic activities depending on the E2 and *Ginkgo* concentration, via ER-dependent and ER-independent pathways [34]. *Ginkgo* reduced the E2 levels by stimulating the E2 metabolism and inhibiting E2 synthesis [34]. As BPH usually is accompanied with inflammation [12, 14], results apparently pointed to the rescuing effect of these three therapies. 

Clinical studies have revealed a close relationship between inflammation and prostate disease [12, 14]. Much of literatures showed more than 92% incidences of inflammatory lesions in prostate tissue in BPH [15, 16]. IL-1 is a paracrine inducer of FGF7, a key epithelial growth factor in BPH [35]. In BPH, IL-1 can be upregulated to induce FGF7, which in turn leads to further epithelial growth and increases IL-1 secretion, establishing the so-called “Double Paracrine Loop” [35]. *Ginkgo*, Ex, and *Ginkgo* + Ex all effectively alleviated the elevation of IL-1 (Figure 7). As well known, such anti-inflammatory effect has been well cited [21–24, 31].

Level of PCNA was lowered in *Ginkgo* and Ex groups comparing to the BPH control. Conversely, the PCNA level in *Ginkgo* + Ex remained unaffected at a level as high as the BPH control (Figure 8(b)).

Literature indicated that the pathogenesis of BPH could be caused by high proliferating rate and low apoptosis rate of hyperplasia epithelium [36]. Recent studies by Alonso-Magdalena et al. demonstrated BPH is not a disease of prostatic stroma proliferation but rather of accumulation of mesenchymal-like cells derived from the prostatic epithelium and the endothelium [9]. No evidence of proliferation was found in the stroma but in the epithelium of some ducts; 0.7% of the basal and 0.4% of the luminal cells were positive for the nuclear antigen Ki67 and the PCNA [9].

**Figure 9:**
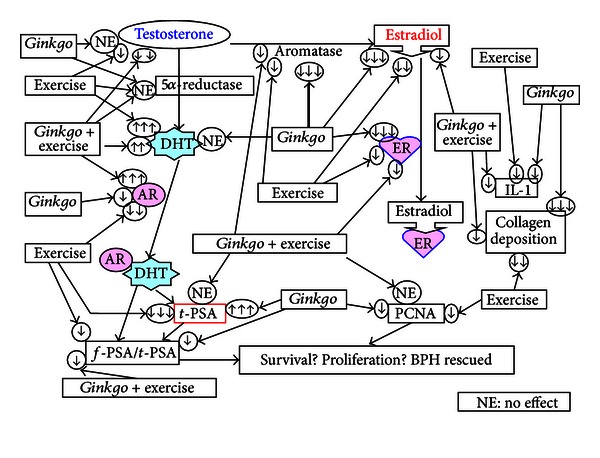
Summary of the therapeutic effects assessed from different treatments: *Gingko*, Ex, and the combined *Gingko* + Ex on the BPH. AR: androgen receptor, ER: estrogen receptor, *f*-PSA: free PSA, *t*-PSA: total PSA, PCNA: proliferating cell nuclear antigen.

In summary, BPH tended to retard growth and increase organ weights of prostate. Although *Ginkgo*, exercise, and *Ginkgo* + Ex were all effective in alleviating collagen deposition, the combined *Ginkgo* + Ex was the most ineffective. Testosterone was unaffected by *Ginkgo* but significantly suppressed by Ex and *Ginkgo* + Ex. The levels of 5*α*-reductase were totally unaffected by all three treatments. *Ginkgo* alone did not affect DHT level, but Ex and *Ginkgo* + Ex highly stimulated DHT. *Ginkgo* and Ex downregulated AR, but *Ginkgo* + Ex highly upregulated the level to higher than the BPH control. The upregulated E2 was suppressed by all three therapies. The highly upregulated PCNA in BPH was significantly and separately suppressed by *Ginkgo* and Ex, but unalleviated by *Ginkgo* + Ex. Results that underlie both *Ginkgo* and exercise were effective, but the combined therapy was ineffective for BPH treatment. The action mechanisms of *Ginkgo* and exercise in treating BPH are summarized in Figure 9.

## 5. Conclusion

In BPH, *Ginkgo* acts as an antiandrogenic (regarding the DHT level), an anti-AR, an aromatase inhibitor, and a potent anti-ER and a strong antiestrogenic (regarding the estradiol level). Exercise acts as an androgenic (regarding the DHT level), an anti-AR, a moderate anti-ER, a moderate aromatase inhibitor, and an estrogenic (regarding the estradiol level). The combined therapy behaves as an androgenic (regarding the DHT level), an AR-upregulator, a moderate anti-ER, a moderate aromatase inhibitor, and an antiestrogenic (regarding the estradiol level). Thus, the therapeutic outcomes could be very complicated and different from each other. Overall, pathologically *Ginkgo *alone and exercise alone may be more beneficial than the combined therapy.
